# Function of Membrane Rafts in Viral Lifecycles and Host Cellular Response

**DOI:** 10.1155/2011/245090

**Published:** 2011-12-07

**Authors:** Tadanobu Takahashi, Takashi Suzuki

**Affiliations:** Department of Biochemistry, School of Pharmaceutical Sciences, and Global COE Program for Innovation in Human Health Sciences, University of Shizuoka, Shizuoka 422-8526, Japan

## Abstract

Membrane rafts are small (10–200 nm) sterol- and sphingolipid-enriched domains that compartmentalize cellular processes. Membrane rafts play an important role in viral infection cycles and viral virulence. Viruses are divided into four main classes, enveloped DNA virus, enveloped RNA virus, nonenveloped DNA virus, and nonenveloped RNA virus. General virus infection cycle is also classified into two sections, the early stage (entry process) and the late stage (assembly, budding, and release processes of virus particles). In the viral cycle, membrane rafts act as a scaffold of many cellular signal transductions, which are associated with symptoms caused by viral infections. In this paper, we describe the functions of membrane rafts in viral lifecycles and host cellular response according to each virus classification, each stage of the virus lifecycle, and each virus-induced signal transduction.

## 1. Introduction

Relationships between virus infection mechanisms and lipid rafts had often been studied in complexes with caveolae [1, 2]. Lipid rafts, membrane microdomains enriched in cholesterol, and sphingolipids represented by GM1 and globotriaosylceramide (Gb3Cer) were defined at the Keystone Symposium on Lipid Rafts and Cell Function (March 23–28, 2006 in Steamboat Springs, CO) as follows: “Membrane rafts are small (10–200 nm), heterogeneous, highly dynamic, sterol- and sphingolipid-enriched domains that compartmentalize cellular processes. Small rafts can sometimes be stabilized to form larger platforms through protein-protein and protein-lipid interactions.” This definition led to the term “lipid raft” being discarded in favor of the term “membrane raft”. The term “membrane raft” underlies the concept that both proteins and lipids, rather than solely lipid-driven interactions, play an important role in the formation of these membrane microdomains. The caveola, a cholesterol/sphingolipid-rich small pit, depression, or invagination, is a site on the cell surface that provides a platform for proteins and lipids to interact and transmit signals. In the symposium, the range of 10–200 nm, which was adopted as the size of membrane rafts, included the upper limit on the surface of a caveola. Here, membrane rafts include caveolae [3].

**Figure 1:**
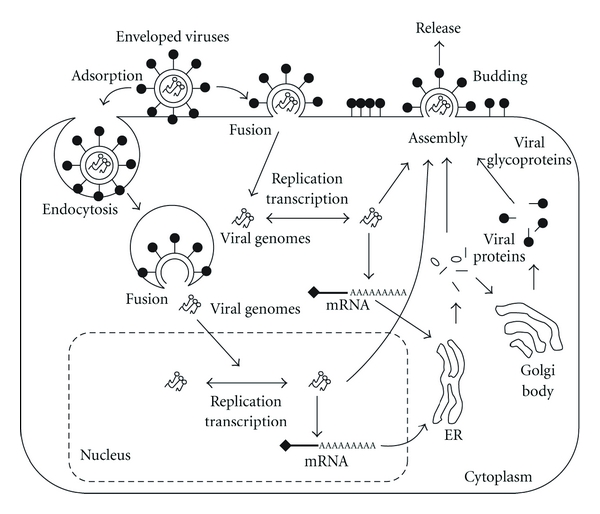
Entry, assembly, and budding processes of enveloped viruses. Some enveloped viruses, such as *Orthomyxoviridae, Rhabdoviridae,* and *Togaviridae *family viruses, are incorporated into cells through an endocytic pathway. Other enveloped viruses, such as *Paramyxoviridae* family viruses, are incorporated into cells through direct fusion between the viral membrane and cell surface membrane. *Herpesviridae* family viruses utilize both pathways. Viral genomes of enveloped RNA viruses, such as *Orthomyxoviridae* family viruses, and enveloped DNA viruses, such as *Herpesviridae* family viruses, are replicated and transcribed in the nucleus. On the other hand, viral genomes of enveloped RNA viruses, such as *Paramyxoviridae* family viruses, are replicated and transcribed in the cytoplasm. After assembly of viral proteins and genomes, progeny viruses are budded and then released from the cell surface membrane.

**Figure 2:**
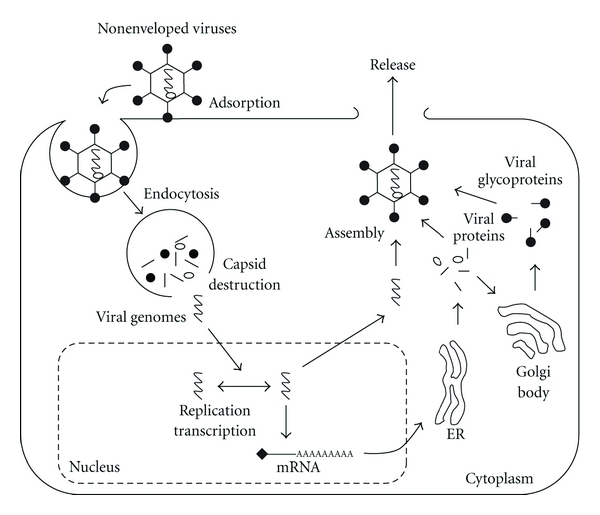
Entry, assembly, and budding processes of nonenveloped viruses. Nonenveloped DNA viruses, such as *Adenoviridae* and *Papovaviridae* family viruses, are incorporated into cells through endocytosis and then their viral DNA genomes are released into the cytoplasm by viral capsid destruction. Viral genomes are subjected to replication and transcription in the nucleus. After assembly of viral proteins and genomes, progeny viruses are released from cells.

Membrane rafts have been shown to be involved in the virus entry, assembly, or/and budding process in infection lifecycles of various viruses, such as retroviruses (*Retroviridae*), RNA viruses (*Arenaviridae*, *Astroviridae*, *Bunyaviridae*, *Caliciviridae*, *Coronaviridae*, *Filoviridae*, *Flaviviridae*, *Orthomyxoviridae*, *Paramyxoviridae, Picornaviridae*, *Reoviridae*, *Rhabdoviridae,* and *Togaviridae*), and DNA viruses (*Adenoviridae*, *Hepadnaviridae*, *Herpesviridae*, *Papovaviridae*, *Parvoviridae, *and *Poxviridae*). These studies have demonstrated the localization of viral structural proteins in membrane rafts and the effects of raft-disrupting agents, which mainly remove cholesterol from the surface membrane or inhibit the synthesis of cholesterol, on the infection and replication processes of these viruses. The virus infection process begins with attachment of the virus to specific receptors on host cell surfaces. Some RNA viruses, such as *Orthomyxoviridae, Rhabdoviridae* and *Togaviridae *family viruses, and DNA viruses, such as *Adenoviridae* and *Papovaviridae* family viruses, enter cells through an endocytic pathway and inject viral proteins and genes directly into the cytoplasm by fusion of the viral envelope with the host cellular membrane or destruction of viral capsids. Other enveloped viruses, such as *Paramyxoviridae* family viruses, allow the viral membrane to fuse directly with the host cell surface membrane and inject the viral proteins and genes directly into the cytoplasm. Transcription and replication of DNA viruses except poxviruses generally proceed inside the nucleus, whereas those of RNA viruses except influenza virus proceed in the cytoplasm. Newly synthesized progeny viral components are transferred to organelles or the plasma membrane, resulting in formation of progeny virus particles by assembly and/or budding. Virus particles are classified by configuration of the viral outer envelope into two types, enveloped viruses (*Arenaviridae*, *Bunyaviridae*, *Coronaviridae*, *Filoviridae*, *Flaviviridae*, *Herpesviridae*, *Hepadnaviridae*, *Orthomyxoviridae*, *Paramyxoviridae Poxviridae*, *Rhabdoviridae* and *Togaviridae*) and nonenveloped viruses (*Adenoviridae*, *Astroviridae*, *Caliciviridae*, *Picornaviridae*, *Papovaviridae*, *Parvoviridae* and *Reoviridae*) (Figures 1 and 2). The envelope constructs of enveloped viruses are lipid bilayers derived from the host cellular membrane of the cell surface, Golgi body, or endoplasmic reticulum (ER), where the viruses are budded. Viral surface proteins transferred to the cell surface are buried in the viral envelope together with the lipid bilayer of the host cell surface (Figure 1). Nonenveloped viruses are generally assembled in the cytoplasm or nucleus and burst into the extracellular environment through membrane destruction from cell death (Figure 2). In this paper, recent findings on the function of membrane rafts in viral lifecycles and host cellular response are discussed.

## 2. Role of Membrane Rafts in Virus Entry

The involvement of membrane rafts in virus entry has been evaluated by the effects of raft-disrupting reagents on virus infection and by the effects of cholesterol-removing reagents such as methyl-*β*-cyclodextrin and cholesterol synthesis inhibitors such as nystatin. Inhibition of virus infection by cholesterol depletion is generally recovered by the addition of exogenous cholesterol without affecting virus binding to cellular receptors. Traditional examinations of membrane raft function in virus entry have been performed by biochemical methods for detection of viral proteins and viral cellular receptors within a detergent-insoluble fraction after virus attachment and during virus infection or colocalization of these proteins and receptors with specific raft markers such as caveolin-1, flotillin, and GM1. Molecular biological examinations of the role of a caveola-dependent endocytic pathway in virus entry have also been performed by inhibition of caveola formation using RNA interference (RNAi) and dominant-negative protein expression for normal caveolin-1 knockdown.

## 3. Entry of Enveloped Viruses

Entry processes of enveloped viruses associated with membrane rafts have been evaluated for lymphocytic choriomeningitis virus (LCMV; *Arenaviridae*) [4], coronaviruses including severe acute respiratory syndrome coronavirus (SARS-CoV) (*Coronaviridae*) [5–11], Ebola virus (*Filoviridae*) [12–14], Marburg virus (*Filoviridae*) [12, 15], West Nile virus (WNV; *Flaviviridae*) [16], dengue virus (DEN; *Flaviviridae*) [17–20], Japanese encephalitis virus (JEV; *Flaviviridae*) [19, 21], human hepatitis C virus (HCV; *Flaviviridae*) [22, 23], Epstein-Barr virus (EBV; *herpesviridae*) [24, 25], herpes simplex virus-1 (HSV-1; *herpesviridae*) [26, 27] including porcine herpesvirus-1 pseudorabies virus [28], human herpesvirus-6 (HHV-6; *Herpesviridae*) [29], human herpesvirus-8 of Kaposi's sarcoma-associated herpesvirus (HHV-8; *Herpesviridae*) [30, 31], influenza virus (*Orthomyxoviridae*) [32–37], vaccinia virus (*Poxviridae*) [38], human immunodeficiency virus (HIV; *Retroviridae*) [39–45], human T lymphotropic virus 1 (HTLV-1; *Retroviridae*) [46, 47], Semliki Forest virus (*Togaviridae*) [48–51], and Sindbis virus (*Togaviridae*) [51, 52].

The majority of enveloped viruses release viral internal genomes and proteins into the intracellular compartment through fusion processes induced by viral surface proteins between viral and cellular membranes immediately after virus attachment to receptors or the endocytic pathway.

Human coronavirus, a pathogen causing 10–30% of all common colds manifesting upper respiratory and gastrointestinal symptoms, enters cells through caveola-dependent endocytosis after attachment to the viral receptor CD13 within membrane rafts. It has been shown that virus infection is inhibited by caveola destruction using a cholesterol-removing reagent and an RNAi method for caveolin-1 [5]. Mouse hepatitis virus (MHV) of murine coronaviruses binds to nonraft membranes but moves to raft membranes for virus entry and fusion processes. Membrane rafts are not incorporated into the MHV virion and are not associated with the spike (S) protein of MHV on the Golgi body membrane, which is the site of virus assembly and budding. Membrane rafts are therefore not required for the virus release process [6, 7]. SARS-CoV, the most extensively researched human coronavirus that causes severe acute respiratory syndrome (SARS), associates with membrane rafts as an entry platform via the viral spike (S) protein after binding to the viral receptor angiotensin-converting enzyme 2 (ACE2) within rafts of Vero E6 cells [8, 10]. Moreover, SARS-CoV and feline infectious peritonitis virus (FIPV), a coronavirus causing lethal chronic disease in cats, enter cells through a clathrin-independent and caveola-independent pathway because both dominant-negative protein expressions of Eps15 (required for the clathrin-dependent pathway) and caveolin-1 have no effect on virus infectivity and no colocalization of caveolin-1 is observed with viral proteins during virus entry. However, inhibition of virus infection by cholesterol depletion indicates the importance of raft-mediated endocytosis for the entry process of these viruses [9, 11]. Taken together, results of studies indicate that raft-mediated endocytosis associated with cholesterol is distinctly different from caveolin-1-dependent endocytosis.

Ebola virus and Marburg virus, two of the most pathogenic viruses in humans and nonhuman primates that cause severe hemorrhagic fever with mortality rates reaching 90%, enter host cells through folate receptor-*α* (FR*α*) as a viral coreceptor, which is a glycosyl phosphatidylinositol-(GPI-) anchored protein within membrane rafts [53]. The severe pathogenesis of these viruses in humans makes studies on the infection mechanism difficult to perform using intact viruses. Results of studies using pseudotypes possessing these viral proteins suggest that filoviruses utilize acidified endosomes for vial entry [54, 55]. Inhibitory effects of a cholesterol-removing reagent and a cholesterol synthesis inhibitor on viral infectivity have demonstrated the requirement of membrane rafts in filovirus entry [12, 15]. The entry process of pseudotypes and virus-like particles bearing glycoproteins of Zaire Ebola virus and Lake Victoria Marburg virus appears to occur through multiple pathways including dynamin-dependent (clathrin-dependent and caveola-dependent endocytosis) and dynamin-independent pathways such as macropinocytosis that is enhanced by Axl, a plasma membrane-associated Tyro3/Axl/Mer (TAM) family member, although Axl is not a receptor for filoviruses [14]. Ebola fusion peptide, a hydrophobic helical domain that belongs to the GP2 membrane fusion protein of Ebola virus, is sensitive to interaction with membrane rafts, suggesting the involvement of membrane rafts in the fusion process of Ebola virus during entry into cells [13].

Flaviviruses enter cells through receptor-mediated endocytosis and are delivered into acidic endosomes, leading to release of viral genomic RNA into the cytoplasm by uncoating of the virion. Severe disease caused by WNV infection is manifested as neurological symptoms such as meningitis, encephalitis, and poliomyelitis. A nonpathogenic laboratory strain of WNV (Sarafend strain) binds to *α*_V_ *β*_3_ integrin as a viral receptor and enters cells through a clathrin-dependent endocytic pathway and activation of focal adhesion kinase (FAK) [56, 57]. However, NY385-99 strain of WNV utilizes a raft-mediated endocytic pathway that is not associated with *α*_V_ *β*_3_ integrin and FAK activation [16]. Use of membrane rafts in the entry process depends on the virus strain.

DEN, an important arthropod-borne human pathogen, is clinically manifested from a simple self-limited febrile illness known as dengue fever to a hemorrhagic fever and potentially fatal hemorrhagic shock syndrome. Reported candidates of the DEN receptor for the entry process include dendritic cell-specific ICAM 3-grabbing nonintegrin (DC-SIGN) [58], heparan sulfate [59], heat shock proteins (HSPs) [18], and neolactotetraosylceramide [60]. HSP90 and HSP70 are part of a receptor complex of DEN and are associated with membrane rafts essential for virus entry into neuroblastoma cells and human monocytes/macrophages [18, 19]. DC-SIGN is present in membrane rafts [61], and neolactotetraosylceramide is a sphingolipid within membrane rafts. One of risk factors of hemorrhagic fever and fatal hemorrhagic shock syndrome after secondary infection with a serotype different from the primary infection is probably antibody-dependent enhancement (ADE), which can be explained by the hypothesis that preexisting nonneutralizing antibodies will generate immune complexes with a new serotype secondary-infecting DEN, leading to enhancement of the capacity to infect macrophages and other Fc*_*Υ*_* receptor-(Fc*_*Υ*_-*) bearing cells. The ADE of DEN-4 infection in the human myelomonocyte cell line U937 has been suggested to require membrane rafts [20]. The entry process of JEV, the leading cause of acute encephalitis in South-East Asia, also requires membrane rafts in neural stem/progenitor cells [19, 21], possibly via the putative receptor HSP70 [62] and heparin sulfate [63] like DEN. The phosphatidylinositol-3 kinase (PI3K)/Akt pathway is activated through membrane rafts as a signal platform during early JEV infection, resulting in increased production of the infectious progeny viruses [21].

HCV is a major cause of chronic liver disease, including chronic hepatitis, hepatic steatosis, cirrhosis, and hepatocellular carcinoma. A cholesterol-removing reagent has an inhibitory effect on HCV entry through the virus receptor CD81 within membrane rafts [22]. Sphingomyelin hydrolysis shows a strong inhibitory effect on HCV entry, because ceramide enrichment of the plasma membrane leads to a decreased level of CD81 at the cell surface membrane by enhancement of CD81 internalization [23]. Thus, the entry process of HCV through CD81 is partially dependent on some major raft components, such as cholesterol and sphingomyelin, at the cell surface membrane.

EBV is a human herpesvirus causing infectious mononucleosis and is associated with a variety of human lymphocytic and epithelial neoplasms, including Burkitt's lymphoma and nasopharyngeal carcinoma. EBV can recognize human complement receptor type 2 (CR2), also known as CD21, on the cell surface membrane of B lymphocytes [24, 64]. Complexes of CD21 with CD19 and palmitoylated CD81 are present in membrane rafts. CD19/CD21/CD81 complexes in part stabilize B-cell antigen receptor (BCR) within membrane rafts, thus leading to enhancement of BCR-mediated signaling [25].

HSV, belonging to the family of alphaherpesvirus, is a pathogen causing mucosal lesions of the mouth and genital organs in humans. HSV binds to and enters host cells through complicated processes involving the essential viral glycoproteins B (gB), gD, gH, and gL and multiple cellular molecules including the tumor necrosis factor receptor (TNFR) family [65], nectin-1 or nectin-2 (two members of the immunoglobulin superfamily) [66], paired immunoglobulin-like type 2 receptor (PILR) [67], and a particular type of modified HSPGs [68, 69]. The association of TNFR with membrane rafts is essential for tumor necrosis factor alpha-(TNF*α*-) mediated nuclear factor-kappaB (NF-*κ*B) activation [70]. Similarly, the association of HSPG with membrane rafts is correlated with protein kinase C alpha (PKC*α*) activation [71]. Nectin-1 binding to gD of HSV-1 is not associated with membrane rafts either before or during HSV-1 infection in several cell lines. In the absence of *α*_V_ *β*_3_-integrin, HSV-1 utilizes raft-independent and dynamin2-independent pathways to reach the intracellular neutral pH compartment, where the viral envelope fuses with the plasma membrane [27]. The gB, but not gC, gD, or gH, of HSV-1 is associated with membrane rafts after virus attachment and during virus entry. Moreover, a cholesterol-removing reagent results in inhibition of HSV-1 and pseudorabies virus entry [26, 28]. These findings indicate that gB may interact with a cellular molecule within membrane rafts that may serve as a platform for HSV-1 entry and cell signaling. Also, *α*_V_ *β*_3_-integrin expression on nectin-1-positive cells allows HSV-1 to enter cells through a raft-dependent and dynamin2-dependent pathway and reach the intracellular acidic compartment, where the viral envelope fuses with the plasma membrane. The pathway dictated by *α*_V_ *β*_3_-integrin utilizes membrane rafts, the platforms for a number of Toll-like receptors, suggesting that *α*_V_ *β*_3_-integrin functions as a sentinel of innate immunity [27].

HHV-6, a betaherpesvirus related to human herpesvirus 7 and human cytomegalovirus, is a human pathogen of emerging clinical significance. Human CD46, a cellular receptor for HHV-6, is not associated with membrane rafts in uninfected cells. However, after virus attachment, CD46 is localized to membrane rafts. When membrane rafts are disrupted by a cholesterol-removing reagent or rescued by addition of exogenous cholesterol, the entry process of HHV-6 is inhibited or partially recovered, respectively. Membrane rafts appear to play an important role in the HHV-6 entry process as an entry site on the host cell surface [29].

HHV-8, the most recently identified member of human gammaherpesviruses, is consistently identified in all forms of Kaposi's sarcoma, primary effusion lymphoma, and multicentric Castleman's disease. Reduced HHV-8 infection and decreased cellular signals associated with the virus infection by raft disruption suggest that membrane rafts in microvascular dermal endothelial cells are required for HHV-8 infection and gene expression, due to their potential roles in the modulation of HHV-8-induced PI3K, RhoA-GTPase, and Diaphanous-2 (a RhoA-GTPase-activated adaptor molecule involved in microtubule activation) signal molecules, which play roles in virus entry processes after receptor binding [30]. Although activation of these signals involved in actin dynamics plays an important role in the entry process and endosomal sorting of HHV-8, the virus enters cells mainly through a clathrin-dependent pathway, but not a raft-dependent pathway, in endothelial cells [31].

Influenza viruses, highly transmittable pathogens of severe acute respiratory symptoms in various animals including humans, avians, and swines, enter host cells through multiple pathways including clathrin-independent endocytosis, caveola-independent endocytosis, and macropinocytosis depending on the cell type [33, 35–37] after binding of a viral envelope glycoprotein, hemagglutinin (HA), to glycoconjugates containing sialic acid on the cell surface [72, 73]. The viruses carried to late endosomes acquire fusion activity of HA given by its low-pH-dependent conformation change, leading to membrane fusion between the virus and endosomes. The viral ribonucleoprotein (RNP) complexes including the viral RNA genome are then released to the cytoplasm of host cells by proton influx of viral ion channel M2 protein that requires binding with cholesterol [32, 74]. HA concentration to membrane rafts provides a sufficient amount of HA for the progeny virus envelope so that it can express efficient fusion activity for cellular membranes [34]. Influenza virus and capsid-like core particles of hepatitis B virus (*Hepadnaviridae*) can also internalize through clathrin-dependent endocytosis alone without the use of membrane rafts [75].

Vaccinia virus had been established as a vaccine that eradicated smallpox disease. Immediately after virus infection, the viral envelope proteins A14, A17L, and D8L, but not H3L, are present in membrane rafts on the cell surface. Initial attachment of the virus to viral receptor glycosaminoglycans is not required for such membrane raft formation. On the other hand, cholesterol-containing membrane raft formation with these viral envelope proteins is observed when vaccinia virus penetrates into a wide variety of mammalian cells from different hosts [38].

HIV-1, a pathogen causing long-term and chronic disease that gradually progresses to acquired immunodeficiency syndrome, binds to viral receptor CD4 on the cell surface by the viral envelope glycoprotein gp120. Conformational change of gp120 after receptor binding leads to interaction with viral coreceptors, chemokine receptor CXCR4 or CCR5, and a subsequent conformational change of the viral envelope glycoprotein gp41 that confers membrane fusion activity [76–78]. Approximately 11–18% of CCR5 in human adenocarcinoma cells, 90–95% of CD4 in H9 leukemic T cells, and 50–66% of CD4 in peripheral blood mononuclear cells (PBMCs) are detected in membrane rafts [79, 80], whereas CXCR4 is almost entirely absent in membrane rafts of human embryonic kidney 293T cells, H9 leukemic T cells and PBMCs [39, 80]. However, recent research indicates that the viral clustering of coreceptor CXCR4 in membrane rafts on 293T cells (not human glioma NP2 and human rhabdosarcoma TE671), rather than that of the viral receptor CD4, is the key step for the entry process of HIV-1 [44]. Interactions between CD4 and CCR5, which occur outside membrane rafts, have been postulated to influence susceptibility to the entry process of CCR5-tropic HIV [81]. A recent study has demonstrated that CD4 and CCR5 in macrophages are partitioned to membrane rafts and has suggested that macrophage membrane cholesterol is required for the entry process of HIV, implicating involvement of membrane rafts [43]. Additionally, glycosphingolipids abundantly present in membrane rafts of host cells, such as Gb3Cer, GM3 gaglioside, and galactosylceramide, have been shown to be involved in the interaction with viral glycoproteins and in the virus entry process [45, 82–85]. The presence of Gb3Cer within membrane rafts in glomerular cells, but not tubular cells, may play a role in HIV nephropathy through binding of gp120 [86]. However, HIV-1 entry into primary human brain microvascular endothelial cells appears to be a raft-independent mechanism associated with proteoglycans such as cell-associated heparin sulfate and chondroitin sulfate [87].

HTLV-1, an oncogenic pathogen causing human adult T cell leukemia, enters host cells through glucose transporter 1 (GLUT-1) [88] that is targeted to membrane rafts for glucose deprivation [89]. Inhibition of vial entry and syncytium formation of the infected cells by a cholesterol-removing reagent suggests the involvement of membrane rafts in the entry and fusion processes of HTLV-1 [46, 47].

Alphaviruses such as Semliki Forest virus and Sindbis virus, arthropod-borne pathogens of infectious arthritis and rashes being the most commonly observed, require cholesterol for the virus entry process and especially for the membrane fusion process between the virus and the endosome triggered by low pH of acidic endosomes [48, 49, 52]. Direct binding of E1 fusion protein of Semliki Forest virus to cholesterol promotes viral fusion and infection in a cholesterol-dependent manner, unlike flaviviruses such as DEN and yellow fever virus, which show no significant binding of viral fusion proteins to cholesterol [90]. However, alphaviruses may not require membrane rafts for cholesterol-dependent promotion of fusion with target membrane [51]. Similarly, the entry process of lymphocytic choriomeningitis virus (LCMV; *Arenaviridae*) is also known to be raft independent but to require membrane cholesterol [4]. Cholesterol dependence may not necessarily be linked to the function of membrane rafts for the virus entry process.

## 4. Entry of Nonenveloped Viruses

Entry processes of nonenveloped viruses associated with membrane rafts have been investigated for species C human adenovirus (HAdV; *Adenoviridae*) [91, 92], Coxsackie virus A9, B3, and B4 (*Picornaviridae*) [93–96], echovirus types 1 and 11 (*Picornaviridae*) [97–102], enterovirus (*Picornaviridae*) [98], Rhinovirus (*Picornaviridae*) [103], BK virus (*Papovaviridae*) [104–106], bovine papillomavirus (*Papovaviridae*) [107], human papillomavirus (HPV; *Papovaviridae*) [108–116], JC virus (*Papovaviridae*) [117], simian virus 40 (SV40; *Papovaviridae*) [118–126], bluetongue virus (*Reoviridae*) [127], and Rotavirus (*Reoviridae*) [128–130].

HAdV, a common pathogen of acute respiratory disease and epidemic keratoconjunctivitis, is frequently used as viral vectors for gene therapy, most of which are serotype 5 that generally utilize a clathrin-dependent endocytic pathway. Initial interaction of HAdV with the cellular coxsackievirus and adenovirus receptor (CAR) and heparin sulfate glycosaminoglycans [131] is followed by interaction of the RGD motif of the virus with *α*_V_ *β*_3_, *α*_V_ *β*_5_, *α*_M_ *β*_2_, and *α*_5_ *β*_1_ integrins, resulting in clathrin-dependent entry of HAdV into hematopoietic cells. In contrast, mature B-cell plasmocytes and Chinese hamster ovary (CHO) cells, which are CAR-negative cell lines, are permissive to HAdV2, HAdV4, and HAdV5, probably through a clathrin-independent and caveola/raft-dependent endocytic pathway [91, 92].

Coxsackievirus A9 infection is one of the most frequent causes of aseptic meningitis and causes various symptoms such as flaccid paralysis, respiratory disease, and chronic myocarditis. This virus utilizes *α*_V_ *β*_3_ integrin as a viral receptor, glucose-regulated protein 78 (GRP78) as a viral coreceptor, and accessory molecule major histocompatibility complex class I (MHC-I) for the entry process. Receptor molecules *α*_V_ *β*_3_ integrin and GRP78 as well as MHC-I are concentrated as a virus entry site in membrane rafts following virus infection. The relationship between activation of Raf/mitogen-activated protein kinase (MAPK) within rafts during infection and virus entry is unclear [93, 94]. Coxsackievirus B4 is a human pathogen causing insulin-dependent diabetes mellitus, also known as type I diabetes, by progressive destruction of pancreatic *β* cells. Attachment of this virus to viral receptor molecules, CAR and CD55, seems to induce the recruitment of these molecules into membrane rafts. Internalization of Coxsackievirus B4 rapidly to the Golgi apparatus is independent of clathrin and appears to be dependent on membrane rafts. However, it has been suggested that CAR can also follow the clathrin-mediated pathway [95]. Coxsackievirus B3 is a human pathogen causing febrile illness, meningitis, and myocarditis. Coxsackievirus B3 Nancy strain cannot bind to the glycosylphosphatidylinositol-(GPI-) anchored complement regulatory protein decay-accelerating factor (DAF), but RD strain, a DAF-binding derivative of Nancy strain, can. Coxsackievirus B3 RD strain possessing the ability of DAF binding enters polarized human intestinal Coca-2 cells through a caveola-dependent but dynamin-independent pathway that requires DAF-mediated tyrosine kinase signals, whereas entry of this strain into nonpolarized HeLa CCL-2 cells requires dynamin and membrane rafts with CAR but not clathrin or caveolin, indicating that the entry pathway of this virus is dependent on cell type such as polarized and nonpolarized cell lines and that the requirement of membrane rafts differs significantly from that of caveolin for virus entry. Coxsackievirus B3 Nancy strain possessing no ability of DAF binding utilizes an entry mechanism similar to that of the RD strain in HeLa CCL2 cells, suggesting no influence of DAF binding on virus entry into HeLa CCL2 cells [96].

Echovirus type 1 and a number of enteroviruses including echovirus type 11 cause nerve paralysis, cerebral meningitis, respiratory symptoms, and anathema. These viruses utilize *α*_2_ *β*_1_ integrin and DAF on the cell surface as the respective receptors, which induce caveola-dependent and membrane raft-dependent endocytosis [97–101]. However, a recent study has suggested that binding of clustered *α*_2_ *β*_1_ integrin with echovirus type 1 initiates a unique entry pathway that is p21-activated kinase 1 (Pak1), GTP-binding protein Rac1, PI3K, phospholipase C (PLC) and actin-dependent but clathirin and caveolaindependent and that can sort cargo to caveosomes [102].

Rhinoviruses, general pathogens of cold and acute respiratory symptoms, colocalize with ceramide-enriched membrane platforms during infection. Rhinoviruses induce microtubule- and microfilament-mediated translocation of acid sphingomyelinase from an intracellular compartment onto the extracellular leaflet of the cell membrane. The enzymatic activity of acid sphingomyelinase hydrolyzes sphingomyelin to ceramide in the cell membrane, finally leading to the formation of large ceramide-enriched membrane platforms. Genetic and pharmacological prevention of acid sphingomyelinase has shown the involvement of ceramide-enriched membrane platforms in Rhinovirus entry [103]. Although previous studies have shown the existence of many receptors for Rhinovirus entry, ninety percent of human Rhinovirus immunogenic variants use intercellular adhesion molecule-1 (ICAM-1) as a receptor [132–134], which is known to be a component of ganglioside GM1-containing membrane rafts [135].

HPVs are well-established pathogens causing cervical cancer and have also been implicated as pathogens in other epithelial cancers, including head and neck cancers. Over 100 different types of HPV, including types 16, 18, 31, 33, 35, 39, 45, 51, 52, 56, 58, 59, 66, and 68 as high-risk carcinogenic HPVs and types 6, 11, 32, 34, 40, 42, 43, 44, 53, 54, 55, 61, 70, 72, 73, 81, 83, 84, 89, and Pap155 as low-risk HPVs, have been identified. In most cases, type 16 is the primary etiologic agent for anogenital malignancies, particularly cervical cancer [136]. Increased expression of HPV type 18 by addition of cholesterol suggests involvement of the HPV infection cycle in membrane rafts [109]. The entry process of HPV begins with viral binding to specifically modified heparan sulfate proteoglycans (HPSGs), most likely syndecans. In addition, *α*6 integrin and laminin 5 have been suggested to be transient receptors for HPV. Although association of HSPGs with membrane rafts has been shown [71], binding of HPV type 33 pseudovirus to HSPGs is followed by delayed caveola-independent endocytosis [110]. Interestingly, the entry process of HPV types 16 and 58 involves clathrin-dependent endocytosis, whereas that of HPV type 31 involves caveola-dependent endocytosis, indicating that HPVs use distinct routes for entry into COS-7 cells (a monkey kidney cell line) [111]. In human keratinocytes (a natural host cell type of HPVs), remarkably slow entry and initiation of HPV type 31 early infection require both caveolin-1 and dynamin-2 (entry half-time of approximately 14 h), different from fast clathrin-dependent endocytosis of HPV type 16 (entry half-time of 4 h) [114]. Both HPV types 16 and 31 require the acidic compartment of the endosomal pathway, where low pH induces a conformational change in the HPV capsid to promote viral genome uncoating. Binding of and infection with HPV type 16, but not HPV type 31, require HPSGs in human keratinocytes. The different mechanisms of these two HPV types may reflect the distinct binding requirement [115]. However, in COS-7 cells, and 293TT cells (a simian virus 40 large T antigen-transformed human kidney cell line), HPV type 31, like HPV type 16, enters the cells through a clathrin-dependent pathway rather than a caveola-dependent pathway as described above [113]. HPV type 16 also uses a novel endocytic pathway associated with tetraspanins CD63 and CD151 in HeLa cells, independently of clathrin and caveolin [116]. Complexes of tetraspanins CD63 and CD151 with *α*6 integrin and laminin through *β*4 palmitoylation of these tetraspanins induce assembly of cholesterol-associated microdomains that are distinct from membrane rafts [137, 138]. These tetraspanin-enriched microdomains may serve as an entry platform of HPV type 16. The different entry routes for HPV types 16 and 31 might result from different host cell types, such as human keratinocytes, COS-7 cells and 293TT cells, and from dependency of HPV lifecycles on cell differentiation. Although the involvement of distinct endocytic pathways in HPV type-dependent pathogenicity remains unclear, results obtained from human keratinocytes, a natural host cell line of HPVs, may be close to the truth of the entry process. BK virus, a pathogen of an infectious complication termed polyomavirus-associated nephropathy in renal transplant recipients, enters Vero cells and human renal proximal tubular epithelial cells by a slow caveola-dependent and clathrin-independent pathway in a pH-dependent manner [104–106]. JC virus and bovine papillomavirus enter cells through clathrin-dependent endocytosis and are subsequently transported from early endosomes to caveolar vesicles and then carried by a slow caveola-dependent pathway [107, 117].

Caveola-dependent endocytosis has been studied mostly by analysis of cell entry of polyomaviruses represented by of SV40, which causes cancer in some animals through repression of tumor suppressor p53 [139]. After SV40 attaches to MHC-I on the cell surface, caveola and caveolin-1 are recruited to the association site of SV40 [140, 141]. Then virus-incorporated caveola undergoes budding from the cellular membrane after activation of tyrosine kinases, actin skeleton depolymerization, actin tail formation, and dynamin accumulation around the association site [121, 123]. Finaly, caveola carries the virus to the endoplasmic reticulum (ER) along cellular microtubules. SV40 receptor MHC-I is not localized in membrane rafts, but attachment induces association of the virus with caveola [118–122] or ganglioside GM1 that is enriched in membrane rafts as a viral receptor [124, 125]. As for other polyomaviruses, BK virus and JC virus, but not mouse polyomavirus, have been reported to use caveola-dependent endocytosis for the entry process [104–106, 117, 142]. Bluetongue virus-1 can infect baby hamster kidney (BHK) cells through an entry process that is clathrin dependent and cholesterol dependent but requires dynamin [127].

Rotaviruses, pathogens of severe diarrhea in infants and young children, recognize several molecules on the epithelial cell surface, including glycolipids, *N*-glycoproteins, HSC70 protein, and *α*_V_ *β*_3_ integrin localized in membrane rafts [128, 129].

## 5. Role of Membrane Rafts in Virus Genome Replication, Assembly, and Budding

Evaluation of the role of membrane rafts in viral assembly and budding has been performed by examining the inhibitory effect of progeny virus formation and production when membrane rafts are disrupted by a cholesterol-removing reagent or a cholesterol synthesis inhibitor. In cholesterol-depleted infected cells, impaired virus formations and productions are recovered by the addition of exogenous cholesterol. General biochemical methods have also been used to determine whether several viral structural proteins during the process of virus formation and assembly are colocalized with the detergent-insoluble fraction or a representative raft-marker such as caveolin-1, flotillin, or GM1. Raft-dependent virus budding and replication have also been evaluated by examining the inhibitory effect of caveola formation using an RNAi method and dominant-negative protein expression for normal caveolin-1 knockdown. If the raft-association sites have been identified in the viral structural proteins, it is possible to generate mutated viral proteins that do not associate with membrane rafts. Then evaluation of virus assembly and budding in membrane rafts can also be performed by measuring intracellular transport or incorporation rate of these mutated proteins into virus particles or characterization of viruses possessing these mutated proteins generated by established reverse genetics methods. When virus budding of enveloped viruses occurs in membrane rafts, colocalization of the budded virus and a raft marker on the cell surface membrane can be observed by using an electronic microscope.

## 6. Genome Replication, Assembly, and Budding of Enveloped Viruses

The role of membrane rafts in viral RNA synthesis of enveloped viruses has been investigated for hepatitis C virus (HCV; *Flaviviridae*) [86, 143–147], DEN [148, 149], JEV [148], and respiratory syncytial virus (RSV; *Paramyxoviridae*) [150, 151].

Association of HCV nonstructural (NS) proteins with cholesterol-enriched membrane rafts in the Golgi-derived membrane results in the formation of the replication complexes (distinct particle structures of about 0.7 *μ*m in size) for HCV RNA synthesis [86, 143, 144]. A lipophilic long-chain base compound, NA255, has been identified as a small-molecule HCV replication inhibitor from a secondary fungal metabolite. NA255 disrupts the association of HCV NS proteins with membrane rafts by prevention of the de novo synthesis of sphingolipids, major membrane raft components [145]. The sphingomyelin-binding domain of HCV RNA-dependent RNA polymerase is the membrane raft localization domain of viral nonstructural protein NS5B [146]. Since cholesterol-depleted or sphingomyelin-hydrolyzed virus has a defect in cellular internalization but not cell attachment, incorporation of cholesterol and sphingolipid into HCV particles also plays an important role in virus maturation and infectivity. Although newly synthesized structural proteins of HCV localize into membrane rafts on the cellular membrane of infected cells, it is unclear whether these membrane rafts are derived from lipids on the viral particles or not. Alternatively, membrane rafts may serve as a budding site of HCV in the ER [147].

On the cell surface and in the viral RNA replication complexes, membrane rafts are associated with the NS1 of all four DEN serotypes and JEV. Efficient viral RNA replication of flaviviruses requires NS1, which can be found in the cell as a monomer associated with cellular organelle membranes and colocalized with the viral replication complex and as a dimer in a membrane GPI-anchored form colocalized with membrane rafts, and it is also secreted as a hexamer from infected cells [148, 149].

Human RSV is a major pathogen of severe lower respiratory tract disease in infants, children, immunosuppressed individuals, and the elderly. The viral proteins, nucleoprotein (N), phosphoprotein (P), large polymerase subunit (L), matrix protein (M) and M2-1, are located in membrane rafts in virus-infected cells. Viral RNP complexes are formed by interactions of viral genomic RNA with N, P, L, and M2-1 proteins. The association of viral RNP complexes with membrane rafts leads to efficient RNA polymerase activity that may require interaction with cellular factor HSP70 (one of the viral receptor candidates) in a raft-dependent and ATP-dependent manner [150, 151].

The role of membrane rafts in the assembly and budding of enveloped viruses has been investigated in Ebola virus [12, 152], Marburg virus [12], WNV [153], murine cytomegalovirus (MCMV; *Herpesviridae*) [154], HSV [155, 156], HHV-6 [157], influenza virus [34, 40, 158–171], measles virus (*Paramyxoviridae*) [40, 172, 173], Newcastle disease virus (NDV; *Paramyxoviridae*) [174, 175], RSV [150, 151, 176–179], Sendai virus (*Paramyxoviridae*) [180, 181], HIV-1 [40, 182–201], HTLV-1 [202], and vesicular stomatitis virus (VSV; *Rhabdoviridae*) [203, 204].

Ebola virus and Marburg virus use membrane rafts bearing viral glycoproteins as a platform for budding from host cells, in addition to entry. Hence, released virus particles incorporate the raft-associated molecule GM1 but not transferrin receptor, a protein absent from membrane rafts [12]. The matrix protein VP40 of Ebola virus, which plays an essential role in virus assembly and budding, oligomerizes in membrane rafts. The cellular TSG101 protein, a component of the vacuolar protein sorting machinery, actively targets along with VP40 to membrane rafts at the cell surface, by binding of VP40 with a PTAP motif of TSG101. Thus, budding complexes containing VP40 and TSG101 accumulate in membrane rafts to promote virus budding [152].

WNV spreads from the blood stream to the central nervous system and peripheral tissues across endothelial cells. Virus-like particles of highly virulent WNV NY99 6-LP strain are transported from the apical side to basolateral side of endothelial cells as the virus budding site in a raft-dependent manner, whereas those of low-virulent WNV Eg101 strain are hardly transported. Membrane rafts may be involved in a transport pathway for basolateral destinations of WNV within endothelial cells [153].

MCMV (belonging to the betaherpesvirus UL78 family) M78 protein, a 7-transmembrane receptor homologue, traffics to the surface membrane of infected cells, but is rapidly and constitutively internalized through both clathrin-dependent and caveola/raft-dependent pathways. Such an M78 subcellular localization may contribute to the incorporation of M78 into the viral envelope during virus maturation [154].

Six envelope glycoproteins, gH, gL, gQ1, gQ2, g, and gB, of HHV-6 are present in membrane rafts during the course of virus maturation. GM1, a representative raft marker, is incorporated into mature virus particles along with these viral envelope glycoproteins, indicating that HHV-6 may assemble through membrane rafts [157].

The HSV tegument contains the less-abundant virus particle host shutoff (vhs) protein encoded by the HSV late gene UL41, which enhances the turnover of all kinetics of viral mRNA and is likely to be important in the increased removal of immediate-early and early viral transcripts to facilitate the switch to late gene expression. Only a small proportion of total cellular vhs proteins are associated with membrane rafts. Nevertheless, a large proportion of the vhs proteins exist in HSV-containing cytoplasmic organelles, indicating that membrane rafts may correlate with assembly of vhs protein into the tegument [155]. The UL11 and UL51 gene products of HSV are membrane-associated tegument proteins that are incorporated into the HSV envelope during virus maturation. HSV UL11 is associated with cholesterol- and glycosphingolipid-enriched membrane rafts through its posttranslational myristoylation and palmitoylation into the Golgi apparatus, but UL51, which is only palmitoylated, has no association with the membrane rafts. UL11 and UL51 appear to function at different steps in progeny virus maturation [156]. Involvement of membrane rafts in HSV assembly and budding remains to be clarified.

Influenza virus particles consist of vial RNP with two spike envelope glycoproteins, HA and neuraminidase (NA), and ion channel M2 protein on the outer surface and internal M1 protein and nonstructural NS2 protein on the inner surface. Membrane rafts are associated with the transmembrane domains and cytoplasmic tails of HA [162] and NA [162, 163] and with the short transmembrane domains of M2 [74, 169, 205] and NP [164], but not M1. These domains of HA and M2 contain palmitoylated cysteine residues that are required for hydrophobic interactions with lipids and cholesterol of membrane rafts. M2 also possesses a putative cholesterol recognition/interaction amino acid consensus (CRAC) motif in addition to palmitoylation of its amphiphilic helix. The targeting to membrane rafts of M2 requires the palmitoylation but not the CRAC motif [171]. Although the transmembrane domains and the cytoplasmic tails of NA are essential for the association with rafts, there is no evidence that NA contains palmitoylated cysteine residues. The mechanism by which NP associates with rafts remains unknown. HA, NA, NP, and M2 independently use membrane rafts together with each apical targeting signal sequence for the apical sorting process, leading to efficient preferential budding and release of progeny viruses from the apical surface membrane. However, membrane rafts are not necessarily required for apical sorting of these viral proteins, indicating that apical sorting machineries of these viral proteins also exist outside their membrane rafts [158, 160–162, 164]. For example, cellular protein VIP17/MAL, a raft-associated protein, is involved in apical transport of HA in dog kidney MDCK cells [206]. M1, a non-raft protein, is incorporated into membrane rafts through its interactions with cytoplasmic tails of both HA and M2, which facilitate the recruitment of internal viral proteins and viral RNP to the cell surface membrane for efficient virus assembly and budding [162, 207]. Although M1 has been believed to play a critical role in viral assembly and budding [208, 209], recent studies have indicated that HA, NA, and M2, but not M1, are required for assembly and budding of influenza virus particles [167, 169].

GM-95 cells are mutant cells of mouse B16 melanoma that cannot synthesize major glycosphingolipids including gangliosides due to lack of ceramide glycosyltransferase gene, the first enzyme of glucosylceramide series synthesis. GM-95 cells can be infected by influenza A viruses and produce infectious progeny viruses, regardless of the absence of major glycosphingolipids [73]. Since gangliosides are major components of rafts and viral receptors, it has been suggested that they are not absolutely necessary for the influenza virus lifecycle. This suggestion for virus assembly and budding is supported by evidence that infectious progeny viruses can be produced from cells infected with mutant viruses possessing HA and NA that lack the ability to interact with membrane rafts by mutations of their raft-binding domains [34, 163] and that membrane raft disruption enhances virus budding from MDCK cells [168]. How membrane rafts help the influenza virus lifecycle needs to be addressed in future studies. The concentration and clustering of HA and NA in the same membrane rafts facilitate efficient incorporation of these raft-associated viral proteins into the progeny viral membrane during the budding process [34, 163], because progeny virus particles are selectively budded together with membrane rafts from the cell surface [159]. At that time, M2 is distributed in a different type of membrane rafts from those associated with HA and NA or is located in non-raft compartments on the cell surface, resulting in poor incorporation into the progeny viral membrane [165]. On the other hand, a study has shown that M2 interacts with membrane rafts associated with HA dependent on an intact actin cytoskeleton and thereby M2 targets to the raft lipid-enriched zone, the viral budding site on the cell surface membrane, organized by HA [170]. Disruption of membrane rafts results in decreased infectious progeny virus production concomitantly with enhancement of the release total infectious and noninfectious virus particles from infected cells [168]. Taken together, the results of studies indicate that the role of membrane rafts in the influenza virus lifecycle is contribution to an efficient incorporation of raft-associated viral proteins into the progeny viral membrane and pinchingoff of virus particles from the plasma membrane.

In infected cells, the tight association of newly synthesized HA transferred to the cell surface with membrane rafts stimulates cellular Raf/MEK/ERK signaling of the MAPK pathway through PKC*α* activation. MPAK activation induces nuclear export of viral RNP compexes from the nucleus to the cytoplasm, leading to enhanced production of infectious progeny viruses [166]. Our recent study has shown that sulfatide, 3-O-sulfated galactosylceramide, is required for efficient virus replication and that association of newly synthesized HA transferred to the infected cell surface with sulfatide induces nuclear export of viral RNP complexes from the nucleus to the cytoplasm, leading to enhanced production of infectious progeny viruses [210]. Thus, association of HA with sulfatide may trigger MAPK activation that enhances nuclear export of viral RNP complexes. Some studies have shown that existence of sulfatide associated with membrane rafts [211, 212], but lipid composition in the purified influenza virus envelope propagated in embryonated eggs does not contain any acidic glycosphingolipids including sulfatide [162]. Further investigation is needed to determine whether enhanced nuclear export of viral RNP is triggered by raft-associated or non-raft sulfatide.

Measles virus is a pathogen of an acute respiratory disease and causes the death of over one million children each year, principally because of virus-induced immunosuppression of lymphocyte function. Membrane rafts act as a platform of the virus assembly process but not the budding process. The transmembrane domain of the viral surface fusion (F) protein has two palmitoylated cysteines, which probably interact with membrane rafts [213]. The F protein is synthesized as an inactive precursor (F_0_) that is proteolytically cleaved in the trans-Golgi network and thereby converted to the biologically active form, disulfide-linked subunits F_1_ and F_2_. After transport of two mature viral envelope glycoproteins, hemagglutinin (H) and F proteins, to the trans-Golgi network, they are preferentially incorporated into membrane rafts, but the F_0_ precursor is not. Internal structural proteins, M and N, interact with membrane rafts possibly through acylation of saturated chains, regardless of the presence of the two viral envelope glycoproteins. The nonstructural V protein remains excluded from rafts during the course of virus infection. Although the M protein can interact with the cytoplasmic tail of the F protein in H-F complexes, it can also bind to plasma membranes and the N protein, thereafter in turn binding to the viral internal structural proteins, P and L. Eventually, M-RNP complexes (composed of viral internal proteins N, P, and L with viral RNA) interact with the surface membrane through the M protein associated with membrane rafts and with H-F complexes associated with membrane rafts through the F protein, resulting in efficient assembly of measles virus prior to the budding process [172, 173].

 NDV is a highly contagious pathogen of zoonotic bird disease affecting many domestic and wild avian species. The assembly and budding of infectious progeny viruses preferentially occur in membrane rafts that interact with the cortical cytoskeleton [174]. Furthermore, intact membrane raft domains in the infected cells, but not in the virus envelopes, facilitate the proper formation or maintenance of the viral surface hemagglutinin-neuraminidase (HN) and F glycoprotein complexes (required for virus-cell membrane fusion) and the incorporation of the HN-F complexes into the viral surface, leading to the initiation of membrane fusion between the virus and cell [175].

Human RSV is a major cause of severe lower respiratory tract disease in young children, the elderly, and immunosuppressed adults. The viral envelope attachment G protein and the internal M and N proteins of RSV are present in membrane rafts. Caveolin, a raft marker, is present in the RSV envelope. RSV infection induces cellular distribution of phosphocaveolin-1 that is enriched at sites of attachment of the actin cytoskeleton. Therefore, RSV assembly at the plasma membrane is shown to occur in specialized membrane rafts that contain a high content of caveolin [176, 177]. The cytoplasmic tail of F protein plays an essential role in its cellular localization and production of infectious progeny viruses, dependently on interaction of F protein with membrane rafts [178]. Moreover, like the function of HIV-1 Nef [192], RSV infection facilitates production of phosphatidylinositol 4,5-bisphosphate (PIP2) and phosphatidylinositol 3,4,5-triphosphate (PIP3) in the lipid composition of membrane rafts within virus inclusion bodies through lipid kinases containing PI3K. This change plays an important role in the assembly and budding processes of progeny virus [179].

Sendai virus, also known as murine parainfluenza virus type 1, is a highly transmissible pathogen of respiratory tract infection in mice, hamsters, guinea pigs, rats, and occasionally pigs. The two viral envelope proteins, HN protein and F protein, interact with membrane rafts. The viral internal M protein appears to have no direct association with membrane rafts. When mature HN-F complexes specifically interact with M proteins through both the cytoplasmic tail and the transmembrane domain of F protein, the HN-F-M complexes are localized in membrane rafts, where the viral proteins have been suggested to be assembled [180]. However, further study led to the conclusion that the virus assembly complexes found in membrane rafts did not constitute a direct precursor of virus particle budding [181]. Two possible routes, raft dependent and raft independent, seem to be involved in Sendai virus assembly.

HIV-1 uses membrane rafts as a platform for viral assembly and budding [182, 188]. Modification of the N-terminus of HIV-1 Gag protein with myristic acid is required for HIV-1 assembly and budding [214]. Gag protein interacts with the plasma membrane through associations between its myristic acids and membrane rafts, leading to its incorporation into HIV-1 particles as an internal structural protein [188]. During the budding process, Gag-Gag interactions (Gag multimerization) occur through the N-terminus of the viral nucleocapsid (NC domain). Lower-ordered Gag multimerization, but not higher-order Gag multimerization, may enhance or stabilize Gag-membrane and Gag-raft interactions. Whether membrane rafts are responsible for increasing Gag-Gag interaction is unclear [186]. The viral envelope glycoprotein complexes (composed of gp120 and gp41) are incorporated into the HIV-1 envelope together with membrane rafts by interactions of Gag with the cytoplasmic tail of gp41, which stabilize the gp120-gp41 interactions. Palmitoylation in cytoplasmic tails of gp41 is required for interactions with rafts but not for interactions between gp41 and Gag protein. Moreover, although associations of rafts with both gp41 and Gag protein are not essential for HIV-1 assembly, infectious progeny virus production, and surface trafficking of the viral proteins [185, 189, 190], the quantal interaction of Gag with cholesterol-enriched rafts facilitates HIV-1 particle production by enhancement of both Gag-membrane interaction and Gag multimerization [193, 194]. Interactions of Gag protein with Annexin 2 at the PIP2-enriched membrane rafts also increase virus production [196].

The Nef protein encoded by primate lentiviruses facilitates virus replication and thus increases the pathogenic potential of HIV. The myristoylated N-terminus and its neighboring basic arginine and lysine residues of Nef increase viral transcription and HIV-1 infectivity through interactions with GM1- and cholesterol-enriched membrane rafts, where Nef binds to both the plasma membrane and the viral structural proteins and participates directly in formation of the budding scaffold, leading to incorporation of Nef into the virus particles, concomitantly with viral structural proteins [183, 184, 187]. The N-terminus of Nef determines its differential membrane avidity and its preferential incorporation into a specific membrane raft for surface membranes or for subcellular membranes [191], which Nef itself has the ability to regulate by modulating the lipid composition of the virus envelope and host cell membrane rafts through, for example, activation of lipid kinases such as PI3K [192]. HIV-1 release from certain cell lines is enhanced by the viral Vpu protein, which is partitioned into membrane rafts. Transmembrane mutants of the Vpu protein have impaired HIV-1 particle release function due to deficiency of raft association but still maintain the ability to down regulate CD4 [200]. For HIV-1 assembly and budding, membrane rafts are also associated with cellular factors such as human cellular cystidine deaminase APOBEC3G (hA3G), BST-2/CD317/HM1.24 (tetherin), caveolin-1, and tetraspanins. hA3G inhibits infectivity of progeny virus particles by its incorporation into virus particles through its association with membrane rafts [195, 201]. Tetherin also exerts antiviral activity against HIV-1 and other enveloped viruses as an interferon-inducible factor of the innate immune system by inhibition of progeny virus particle release from the cell surface. Viral propagation among T cells proceeds by direct cell-to-cell transmission through membrane raft-enriched synapses colocalized with tetherin, which counteracts the viral Vpu protein partitioned to membrane rafts, involved in virus release [199]. HIV-1 infection enhances the expression of caveolin-1, a major protein of membrane rafts. Although membrane rafts have been shown to contribute to assembly and budding processes of HIV-1, expression of caveolin-1 causes a reduction of virus production in macrophages [198]. This suggests that caveolin-1 may contribute to persistent infection in macrophages and that a caveolin-dependent raft, caveola, is not necessarily an advantage for the HIV-1 lifecycle. A tetraspanin-enriched microdomain is a unique type of protein-based microdomain, clearly distinct from membrane rafts. Tetraspanins CD9, raft-associated CD55, and raft marker GM1 are concentrated at the virus assembly site. This recruitment and confinement of CD9 are partially dependent on cholesterol, whereas those of CD55 are completely dependent on cholesterol. HIV-1 assembly creates specialized microdomains for recruiting components of both membrane rafts and tetraspanin-enriched microdomains [197]. As for retroviruses other than HIV, HTLV-1 assembly, but not budding and surface trafficking, is also inhibited by a decrease of Gag associations with membrane rafts by interferon *α*-2a treatment [202].

VSV budding is known to occur at membrane microdomains containing the viral envelope G glycoproteins, some of which are 100–150 nm in size and smaller than the virus envelope (approximately 100–150 nm) and others of which extended in size to a maximum of 300–400 nm from the tip of the virus budding site [203, 204]. However, immunoelectron microscopy observation did not confirm that gold-labeled G protein-containing microdomains are equivalent to lipid-enriched membrane rafts. Furthermore, such microdomains of 300–400 nm in size are too large for the definition of raft microdomains of 10–200 nm in size [3]. A recent study has also shown that most G proteins of wild-type VSV were not incorporated into membrane rafts in infected osteoclasts [215]. Relevance of membrane rafts on VSV infection will be settled in future studies.

## 7. Genome Replication, Assembly, and Budding of Nonenveloped Viruses

The role of membrane rafts in intracellular assembly of nonenveloped viruses has been investigated only in bluetongue virus [216] and Rotavirus [130, 217–220], belonging to the family *Reoviridae*. Association of SNARE (soluble *N*-ethylmaleimide-sensitive fusion attachment protein receptor) domain in the bluetongue virus outer capsid VP5 with membrane rafts may play an important role in its membrane targeting and virus assembly [216]. Rotavirus replication occurs at large inclusions (known as viroplasms) in the cell cytoplasm, which are sites for replication of viral RNA and assembly of double-layered particles. These particles are transferred across the ER membrane by interactions of the viral capsid protein VP6 with the nonstructural transmembrane glycoprotein NSP4, which has been characterized as an ER intracellular receptor and a viral enterotoxin for intestinal cells. During transfer across the ER, the virus acquires a transient lipid envelope that is finally lost and replaced by viral surface spike proteins, VP4 and VP7. In this process, the involvement of transient enveloped particles in membrane rafts is highly questionable since rafts are thought to be absent from the ER. Association of VP4 with membrane rafts in the extrareticular compartment facilitates rotavirus morphogenesis as a final assembly platform and apical targeting toward the release process [130, 217, 218]. The raft-type membrane microdomains associated with VP4 are significantly dependent on raft heterogeneity of cell lines [219]. Moreover, *N*-glycosylation of NSP4 is processed in the Golgi network through caveola-dependent Golgi network-bypassing transport [220].

## 8. Role of Membrane Rafts in Virus-Induced Signal Transductions

Viral replication efficiency, viral infection sites, and viral infectious diseases are frequently controlled by membrane rafts in host cells and immune cells. Membrane rafts also act as a scaffold of various cellular signal transductions. Involvement of membrane rafts in many viral infectious diseases often results from up- or downregulation of cellular signal transductions associated with cell proliferation, apoptosis, cell differentiation, immune response, and so on.

HTLV-1 Tax1 protein recruits I*κ*B kinases (IKKs) to membrane rafts for persistent activation of NF-*κ*B, which enhances T-cell proliferation, thereby contributing to HTLV-1-induced T-cell leukemia [221].

Flaviviruses DEN type 2 and JEV activate the raft-dependent PI3K/Akt pathway that induces antiapoptosis in order to protect infected cells from early apoptotic cell death. However, this signaling is not essential for flavivirus replication. A balance between apoptotic and antiapoptotic signaling, which is triggered by the interplay between host and virus, controls the outcome of flavivirus infection [17]. DEN NS1, which can be found in membrane rafts on the host cell surface, increases NF-*κ*B transcriptional activities by facilitating nuclear translocation of NF-*κ*B p65 protein in HepG2 cells, suggesting a possible contribution to DEN pathogenicity [149]. In JEV-infected microglia, the integrity of membrane rafts and the activation of Src-related Ras/Raf/ERK cascades participate in NF-*κ*B activation and consequent TNF*α* and interleukin-1 beta (IL-1*β*) expression. These signal activations and cytokine expressions caused by JEV infection may play a critical role in neuronal cell death [222].

The partitioning of measles virus F protein into high buoyant density-membrane rafts activates an alternative pathway of human complement independently of CD46 and CD55, which regulate the complement activation and do not exist in the same rafts as the F protein. Thus, measles virus infection induces inflammatory response through alternative complement activation [223]. Measles virus-induced immunosuppression also results from signal transduction alteration such as PI3K present in membrane rafts of T cells [224].

HSV-1 infection is implicated in Alzheimer's disease susceptibility by virus binding to HSPGs, nectin receptors, *α*2-macrolgobulin, blood-borne lipoproteins, and apolipoprotein E. Cholesterol reduction on the plasma membrane by a cholesterol synthesis inhibitor has been linked to a decrease in the risk for development of Alzheimer's dementia. Since HSV-1 uptake into cells is dependent on cholesterol and membrane rafts, cholesterol reduction may decrease the availability of raft-dependent pathways to spread HSV-1 in the brain [225, 226]. Tyrosine kinase-interacting protein (Tip) of lymphotropic herpesvirus saimiri (HVS) down-regulates T-cell receptor (TCR) and CD4 expression on the cell surface through its targeting to membrane rafts in T cells. Tip is required for cellular membrane deformation of T cells but not for viral replication, which induces lymphoma in primates [227]. HHV-8 encodes two RING finger E3 ubiquitin ligases (MIR1 and MIR2) that mediate ubiquitination and degradation of cellular proteins important for immune response. Many of the MIR substrates are believed to be present in membrane rafts. Function of MIR2 is required for its palmitoylation, which is known as a posttranslational modification that enhances recruitment of transmembrane proteins into membrane rafts. MIR2 function may play an important role in immune evasion of the virus and resultant persistent viral infection by MIR2-mediated down-regulation of MHC-I and platelet endothelial cell adhesion molecule 1 (PECAM-1) [228].

GM1 expression and asialo-GM1 expression in membrane rafts of T cells and natural killer (NK) cells are differentially regulated by these cells in the context of RSV infection. Asialo-GM1 may increase RSV clearance by increasing IFN-*γ* levels in mouse lungs [229].

The pseudorabies virus Us9 protein interacts with membrane rafts and then promotes targeting of viral structural proteins to neuronal axons. Consequently, the virus spreads from presynaptic to postsynaptic neurons and cells of the mammalian nervous systems [108].

Raft-dependent phagocytosis of HCV-infected apoptotic vesicles containing viral double-strand RNA (dsRNA) is required for maturation of human monocyte-derived dendritic cells (MoDCs). However, HCV JFH1 strain, which can efficiently replicate in cell culture, does not directly stimulate MoDCs for activation of T cells and NK cells [230]. HCV envelope E2 protein attenuates interleukin-2 (IL-2) production at the level of secretion by its interaction with tetraspanin CD81 coreceptor and sequent recruitment of protein kinase C beta (PKC*β*), which is essential for IL-2 secretion, to membrane rafts in peripheral blood mononuclear cells. The ability of the E2 protein to attenuate IL-2 and IFN-*γ* secretion has been suggested to contribute to a mechanism for HCV to evade the human immune response and to establish persistent infection [231].

Antibodies against SARS-CoV spike domain2 (S2) in patient sera can cross-react with human lung epithelial cells through annexin A2, which has been identified as one of the candidate proteins of the autoantigen. SARS-CoV-induced cytokines interlekin-6 (IL-6) and INF*γ* stimulate surface expression and raft distribution of annexin A2 in human lung type II epithelial A549 cells and increase the binding capability of anti-S2 antibodies to human lung epithelial cells. The upregulated expression and raft targeting of annexin A2 and the cross-reactivity of anti-S2 antibodies to annexin A2 may contribute to the pathogenesis of SARS disease [232].

Rhinovirus serotype 39 colocalizes with Src kinases, PI3K, and the serine threonine kinase Akt in membrane rafts in the context of virus infection. Src and PI3K are upstream activators of Akt and the interleukin-8 (IL-8) promoter. Rhinovirus infection activates these kinases and sequent IL-8 expression, which exacerbates asthma and chronic obstructive pulmonary disease [233].

Persistent HPV infection results in transformation of epithelial cells that induces cellular polarity disturbance, which is implicated in MAL and BENE, components of the membrane raft's machinery for apical sorting of membrane proteins. Down-regulation of MAL and BENE genes in the context of HPV infection may play an important role in development of human cervical squamous cell cancer [112]. The “early” gene oncoproteins E6 and E7 of high-risk HPV are known to be invariably expressed in cervical cancers by inducing several signal alterations such as p53 inactivation, apoptosis suppression, telomerase activation, and cell adhesion disruption [234]. The additional oncoprotein E5 of high-risk HPV type 16 increases expression and association of both GM1 and caveolin-1, components of membrane rafts, on the cell surface. This up-regulation of membrane rafts helps HPV immune evasion by suppression of cytotoxic T lymphocytes and enhances proliferative signaling of epidermal growth factor (EGF) possibly through localization of the EGF receptor with membrane rafts [235].

## 9. Role of Membrane Rafts in Prion Infection

Involvement of membrane rafts in infectious particle prion (PrP) infection has been reported [236–242]. PrP is an infectious protein that does not have a genome, unlike viruses. PrP^Sc^, the protease-resistant isoform of the host normal prion protein PrP^c^, is the infectious component causing fetal neurodegenerative transmissible spongiform encephalopathies, called Creutzfeldt-Jakob disease in humans. PrP^c^, which contains a conserved N-terminal cationic domain that stimulates a raft-dependent pathway, internalizes in neuroblastoma N2a cells through a clathrin-independent pathway associated with Arf6 [241] and raft-dependent macropinocytosis [240]. On the other hand, PrP^c^ internalizes in Fischer rat thyroid (FRT) cells by cooperation of clathrin-dependent and raft-dependent pathways. This internalization does not affect caveolin expression in FRT cells, which do not express caveolin-1 and do not have any caveolae. These findings indicate that pathways of PrP^c^ internalization are dependent on cell type and that the raft-dependent pathway distinctly differs from the caveola-dependent pathway [242]. Association of PrP^C^ with cholesterol-enriched membrane rafts enhances correct protein folding of PrP^c^ conformation. Cholesterol depletion, but not sphingolipid depletion, leads to abnormal protein folding from PrP^c^ to PrP^Sc^ [236–238]. GPI-anchored HSPG glypican-1 directly interacts with both PrP^c^ and PrP^Sc^ and targets these to membrane rafts. Targeting of both prions through glypican-1 facilitates favorable interaction of PrP^Sc^ with PrP^c^ within membrane rafts, which are believed to be the conversion sites of PrP^c^ to PrP^Sc^, suggesting a critical role of glypican-1 in the pathogenesis of prion disease [243]. PrP^c^ is associated with membrane rafts in membrane-derived microvesicles of human plasma that are important modulators of cell-to-cell communication. Membrane-derived microvesicles bearing PrP^c^ within membrane rafts may contribute to intercellular diffusion, intracellular signaling, and neuroinvasion of PrP^c^ [244]. The property of the GPI anchor attached to PrP^Sc^ has been reported to affect the binding of PrP^Sc^ to neurons, distribution to membrane rafts, and conversion of endogenous PrP^c^ in GT1 neuronal cells [245]. Moreover, the increased level of glycosphingolipid GM1 (an essential raft marker) on fibroblast cells by a mouse parvovirus (*Parvoviridae*) infection may promote prion infection through the incorporation of exogenous PrP^Sc^ into membrane rafts [239].

## 10. Conclusion

Many studies have suggested the involvement of membrane rafts in cell entry, genome replication, assembly, budding, and virus-associated diseases of enveloped and nonenveloped viruses (summarized in Tables 1, 2, 3, and 4). Paradoxically, some recent studies have shown that membrane rafts are not necessarily required for efficient viral replication. For the virus entry process, many viruses use several different pathways, not only caveola/raft-dependent but also clathrin-dependent or another endocytic pathway such as macropinocytosis. Additionally, the caveola-dependent pathway does not necessarily correspond to the raft-dependent one, suggesting the existence of other complicated pathways associated with caveolae and membrane rafts. For the assembly and budding processes of many enveloped viruses, membrane raft disruption on host cells facilitates formation and production of progeny virus particles with less infectivity and lower viral components. Also, membrane rafts are not necessarily essential for cellular membrane targeting of viral structural proteins. For virus assembly and budding, membrane rafts are more likely to provide progeny virus particles with greater infectivity because of the concentration and efficient incorporation of viral structural components from the assembly and budding sites to the virus particles. Similarly, concentration of viral polymerases into membrane rafts acts as a platform of more efficient replication and transcription of viral genomes. Furthermore, the use of several endocytic pathways provides an advantage for virus entry into a wider range of hosts, cell lines, and tissues or can allow the virus to be assigned to an alternative pathway when one pathway does not work. Concentration of viral fusion proteins in raft-associated receptors or caveola/raft-dependent endocytosis may enhance membrane fusion between the virus and cell, leading to efficient release of viral internal proteins and genomes to the cytoplasm at an early stage of virus infection. Taken together, the results indicate that membrane rafts are not essential for viral life cycles. Viruses probably take advantage of membrane rafts for more efficiency of virus entry, viral genome replication, and virus particle production. Viruses also induce many raft-mediated cellular signals which relate to characteristic symptoms of the viral diseases.

Many studies on the involvement of membrane rafts in viral infection cycles and viral infectious diseases have been performed by classical approaches (cholesterol-disrupting reagent treatments, detergent-insoluble fractionization, and microscopic observation of colocalization with raft markers) and by recent molecular biological approaches (RNAi and dominant negative expression against representative raft components). However, since various types of microdomains, such as raft-dependent, caveola-dependent, cholesterol-dependent, and other specialized microdomains, have been shown to have independent heterogeneous properties, evaluation of respective functions of distinct membrane rafts and microdomains would be difficult by experiments using only common approaches to study membrane rafts. Further study will require new approaches for elucidating the functions of distinct membrane microdomains. An understanding of the role of membrane rafts in viral lifecycle may contribute to elucidation of essential cellular functions of membrane rafts and to development of new antiviral chemotherapy against viral replications and viral infectious diseases.

## Figures and Tables

**Table 1:** Function of membrane rafts in enveloped DNA viruses.

Family	Virus	Process
	Epstein-Barr virus (EBV)	Entry
	Herpes simplex virus-1 (HSV-1)	Entry Assembly Progeny virus infectivity Alzheimer's disease susceptibility
	Porcine herpesvirus-1 (pseudorabies virus)	Entry Viral spread in neurons
*Herpesviridae*	Human herpesvirus-6 (HHV-6)	Entry Assembly
	Human herpesvirus-8 (HHV-8)	Entry Viral immune evasion Persistent infection
	Murine cytomegalovirus (MCMV)	Assembly
	Lymphotropic herpesvirus saimiri (HVS)	Lymphoma
*Poxviridae*	Vaccinia virus	Entry Penetration

**Table 2:** Function of membrane rafts in enveloped RNA viruses.

Family	Virus	Process
*Arenaviridae*	Lymphocytic choriomeningitis virus (LCMV)	Entry
*Coronaviridae*	SARS-CoV, coronavirus	Entry Lung pathogenicity
*Filoviridae*	Ebola virus	Entry Fusion Assembly Budding
	Marburg virus	Entry Budding
*Flaviviridae*	Dengue virus (DEN)	Entry Viral RNA replication ADE of viral infection Antiapoptosis Pathogenicity
	Japanese encephalitis virus (JEV)	Entry Viral RNA replication Progeny virus production Anti-apoptosis Neuronal pathogenicity
	West Nile virus (WNV)	Entry Basolateral transportation in endothelial cells Viral spreads from the blood stream to the central nervous system and peripheral tissues
	Human hepatitis C virus (HCV)	Entry Vrial RNA replication Progeny virus infectivity Budding MoDC maturation Viral immune evasion Persistent infection
*Orthomyxoviridae*	Influenza virus	Fusion Assembly Budding Progeny virus infectivity Apical targeting Viral proton channel
*Paramyxoviridae*	Measles virus	Assembly Inflammatory response Immunosuppression
	Newcastle disease virus (NDV)	Assembly Budding Progeny virus infectivity
	Respiratory syncytial virus (RSV)	Viral RNA replication Assembly Budding Host immune response
	Sendai virus	Assembly
*Retroviridae*	Human immunodeficiency virus (HIV)	Entry Assembly Budding Release Progeny virus production Viral nephropathy Persistent infection in macrophages
	Human T lymphotropic virus 1 (HTLV-1)	Entry Fusion Assembly T cell leukemia
*Rhabdoviridae*	Vesicular stomatitis virus (VSV)	Budding
*Togaviridae*	Semliki forest virus Sindbis virus	Fusion Entry

**Table 3:** Function of membrane rafts in nonenveloped DNA viruses.

Family	Virus	Process
*Adenoviridae*	Species C human adenovirus (HAdV)	Entry
		
*Papovaviridae*	Simian virus 40 (SV40)	Entry
	BK virus	Entry
	JC virus	Entry
	Bovine papillomavirus	Entry
	Human papillomavirus	Entry
	(HPV)	Immune evasion Persistent infection Cancer development
*Parvoviridae*	Mouse parvovirus	Prion infection

**Table 4:** Function of membrane rafts in nonenveloped RNA viruses.

Family	Virus	Process
*Picornaviridae*	Echovirus types 1 and 11	Entry
	Enterovirus	Entry
	Rhinovirus	Entry Cellular kinase activation
	Coxsackie virus A9, B3 and B4	Entry
		
*Reoviridae*	Rotavirus	Entry Assembly Apical targeting Golgi transport
	Bluetongue virus	Entry Assembly Membrane targeting
